# Broadband Silicon-On-Insulator directional couplers using a combination of straight and curved waveguide sections

**DOI:** 10.1038/s41598-017-07618-6

**Published:** 2017-08-03

**Authors:** George F. R. Chen, Jun Rong Ong, Thomas Y. L. Ang, Soon Thor Lim, Ching Eng Png, Dawn T. H. Tan

**Affiliations:** 1 Singapore University of Technology and Design, 8 Somapah Road, Singapore, 487372 Singapore; 2 Electronics and Photonics Department, Institute of High Performance Computing, Agency for Science Technology and Research, 1 Fusionopolis Way, Singapore, 138632 Singapore; 3 Optic2Connect (O2C) Pte Ltd, 79 Ayer Rajah Crescent #01-14, Singapore, 139951 Singapore

## Abstract

Broadband Silicon-On-Insulator (SOI) directional couplers are designed based on a combination of curved and straight coupled waveguide sections. A design methodology based on the transfer matrix method (TMM) is used to determine the required coupler section lengths, radii, and waveguide cross-sections. A 50/50 power splitter with a measured bandwidth of 88 nm is designed and fabricated, with a device footprint of 20 *μ*m × 3 *μ*m. In addition, a balanced Mach-Zehnder interferometer is fabricated showing an extinction ratio of >16 dB over 100 nm of bandwidth.

## Introduction

The use of directional couplers as 2 × 2 power splitters / combiners is ubiquitous in photonic integrated circuits. Due to its simplicity and ease of design, it is commonly used in multiplexing circuits^1, 2^, optical switches^3, 4^, polarisation splitters^5^, and wavelength filters^6^. However the directional coupler splitting ratio is known to be very sensitive to the operating wavelength, as can be seen in high index contrast platforms like SOI.

Many different approaches have been explored in order to develop a wavelength insensitive power splitter (see Table 1). Multi-mode interference (MMI) couplers are commonly used as broadband optical power splitters. However, in general, MMI couplers suffer from output imbalance and excess loss^7–9^. Adiabatic couplers are used in modulators and switches to help ensure broadband operation^10–13^. Although they are fabrication tolerant, they are also much longer than directional couplers. Two cascaded directional couplers with an intermediate phase delay section can form a broadband Mach-Zehnder interferometer type coupler, but the device length is increased^3, 14, 15^. Sub-wavelength gratings (SWG) can be used to engineer the dispersion of directional coupler supermodes, and thus increase operational bandwidth^16–18^. However, SWG directional couplers are sensitive to grating pitch and fill-factor variations, and it may be challenging to meet the required minimum feature size. Finally, asymmetric directional couplers, consisting of waveguides possessing different (or varying) core sizes or bending radii, also show wavelength flattened response^4, 19, 20^. Moreover, numerical studies indicate that SOI curved directional couplers have the advantages of being low loss, comparable in size with conventional straight directional couplers, and also being fabrication and temperature tolerant^21^.

**Table 1:** Recent results of TE mode wavelength insensitive 2 × 2 port 3 dB power splitters on SOI platform.

Type	Length × Breadth	3 dB ± 1 dB Bandwidth	Reference
MMI	9 × 3 *μ*m	60 nm	Xu *et al*.^8^
MMI	7 × 3 *μ*m	80 nm^†^	Chen *et al*.^9^
Adiabatic, Rib	165 × 4 *μ*m	94 nm	Yun *et al*.^12^
Adiabatic, Channel	165 × 4 *μ*m	80 nm	Yun *et al*.^13^
Subwavelength Grating, Directional Coupler	14 × 3 *μ*m	100 nm	Wang *et al*.^17^
Subwavelength Grating, Adiabatic	50 × 2 *μ*m	130 nm	Yun *et al*.^18^
MZI	85 × 30 *μ*m	110 nm*	Campenhout *et al*.^3^
MZI, Asymmetric Waveguides	32 × 2 *μ*m	88 nm	Lu *et al*.^15^
Curved Directional Coupler	20 × 3 *μ*m	140 nm*	Chen *et al*.^4^
Curved Directional Coupler, Straight Sections	20 × 3 *μ*m	88 nm	This Work

*Estimated from balanced MZI extinction.

^†^Simulation result.

In this paper, we study in detail the design of curved asymmetric directional couplers, where the asymmetry arises due to different bending radii between the two constituent waveguides of the coupler. We note that coupler waveguides may or may not necessarily have the same core size, which is a further asymmetry. Such curved directional couplers have previously been studied using coupled-mode theory (CMT)^21^. However, it is not sophisticated enough to account for the coupling contributions from the converging/diverging sections at the input and output of the curved waveguide regions^22^. Here, we use a transfer matrix method (TMM) which is able to account for this additional contribution. Moreover, we allow for the addition of straight coupler sections before the central curved coupler section, which acts as an additional tuning parameter to achieve the desired transmission characteristic. We used the TMM to determine the coupler section lengths and curved coupler radii that give the desired wavelength insensitive operation. We focus on a 50/50 power splitting ratio, but in principle a different splitting ratio is also feasible. We fabricated a 50/50 curved coupler of footprint 20 *μ*m × 3 *μ*m, showing a ±1 dB bandwidth of 88 nm centered at 1565 nm. In addition, we fabricated curved couplers and balanced Mach-Zehnder interferometers (MZI) using IME A*STAR MPW (multi-project wafer) service in order to verify our designs with silicon photonics foundry services.

### Design

Curved directional couplers have previously been modeled using CMT as a uniformly asymmetric coupler^19, 20^. The bar and cross port powers at the output of the coupler of length *L* with power input of *P*_1_(0) only at single input is given by

1 $${P}_{1}(L)={P}_{1}\mathrm{(0)}-{P}_{2}(L)$$

2 $${P}_{2}(L)={P}_{1}\mathrm{(0)}\frac{{k}^{2}}{{k}^{2}+{\delta }^{2}}{\rm{s}}{\rm{i}}{{\rm{n}}}^{2}(\sqrt{{k}^{2}+{\delta }^{2}}L)$$

where *k* is the coupling coefficient as determined by an overlap integral of the electric fields^23^, and *δ* = (*β*_1_ − *β*_2_)/2 is the phase mismatch between the two waveguides. As can be seen, the maximum coupled power is limited by the phase mismatch *δ*. By choosing the coefficient *k* ^2^/(*k* ^2^ + *δ* ^2^) to be the desired cross coupled ratio, wavelength insensitive operation is achieved at a length corresponding to the peak of the sine term, which has a gradient of zero and least subject to perturbations. However, the CMT theory approach is only accurate for weakly coupled waveguides and moreover Eq. 1 does not account for the effects of the converging/diverging bends. Below, we use a more general TMM method to model curved directional couplers.

**Figure 1:**
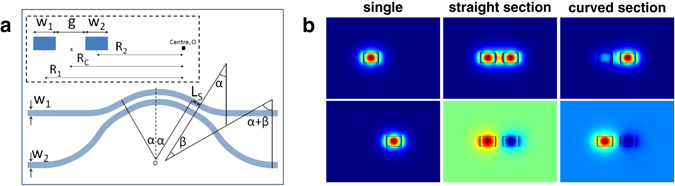
(**a**) SOI curved directional coupler layout and design parameters. (**b**) First and second TE modes at various points along the curved directional coupler.

Figure 1(a) shows the detailed layout parameters of a curved directional coupler. Two waveguides are brought together to a separation distance, gap *g*. Both the waveguides start with an input convergent section, followed by an intermediate straight section and then a central curved section. The input and output sections are symmetric with respect to the centre dotted line of the coupler. We emphasise that the intermediate straight sections (i.e. parallel waveguides) are a generalisation from previously proposed curved directional couplers, which do not possess any straights.

The cross section at the dotted line is fully illustrated in the subset of Fig. 1(a). The main parameters that determine the cross coupling are the radii *R*_1_ and *R*_2_, the coupler gap *g*, the waveguide widths *w*_1_ and *w*_2_, and the coupler curved lengths *L*_*c*_ and coupler straight length *L*_*s*_. *L*_*c*_ is given by *L*_*c*_ = 2*α* · *R*_*c*_. *R*_*c*_ is the distance from the centre O to the midpoint of the gap between the two waveguides. It is given by *R*_*c*_ = *R*_1_ − (*w*_1_ + *g*)/2 = *R*_2_ + (*w*_2_ + *g*)/2. *R*_1_ and *R*_2_ is the distance from centre O to the midpoint of the top and bottom waveguide respectively. *α* and *β* refer to the angles subtended by the curved waveguides shown in Fig. 1(a). Since the angle of the curved coupling section is 2 *α*, it is compensated by *α* to have the waveguide back to straight horizontal. The bottom waveguide is curved with an additional *β* to further the distance away from the top waveguide. Similarly, it is compensated by *α* + β to have it return to straight horizontal. Figure 1(b) shows examples of the first and second TE modes at various points along the coupler, as calculated by a commercial mode solver^24^.

To model the entire curved directional coupler device, one needs to account for the coupling contributions from the input/output edge sections, the straight sections and also the curved sections. We can approximate the edge contributions as straight couplers with an effective length and lump it together with the actual straight length. Using a TMM formalism to describe the propagation through the device, the input and output mode amplitudes of the couplers are expressed as:

2a $$[\begin{array}{c}{A}_{\mathrm{1,}out}\\ {A}_{\mathrm{2,}out}\end{array}]={C}_{ss,s}^{T}\cdot {P}_{ss}\cdot {C}_{cs,ss}^{T}\cdot {P}_{cs}\cdot {C}_{cs,ss}\cdot {P}_{ss}\cdot {C}_{ss,s}[\begin{array}{c}{A}_{\mathrm{1,}in}\\ {A}_{\mathrm{2,}in}\end{array}]$$

2b $${C}_{ss,s}=[\begin{array}{ll}\langle {\hat{e}}_{s\mathrm{,1}},{\hat{e}}_{ss\mathrm{,1}}\rangle  & \langle {\hat{e}}_{s\mathrm{,2}},{\hat{e}}_{ss\mathrm{,1}}\rangle \\ \theta \langle {\hat{e}}_{s\mathrm{,1}},{\hat{e}}_{ss\mathrm{,2}}\rangle  & -\theta \langle {\hat{e}}_{s\mathrm{,2}},{\hat{e}}_{ss\mathrm{,2}}\rangle \end{array}],\,{C}_{cs,ss}=[\begin{array}{ll}\langle {\hat{e}}_{ss\mathrm{,1}},{\hat{e}}_{cs\mathrm{,1}}\rangle  & \langle {\hat{e}}_{ss\mathrm{,2}},{\hat{e}}_{cs\mathrm{,1}}\rangle \\ \eta \langle {\hat{e}}_{ss\mathrm{,1}},{\hat{e}}_{cs\mathrm{,2}}\rangle  & -\eta \langle {\hat{e}}_{ss\mathrm{,2}},{\hat{e}}_{cs\mathrm{,2}}\rangle \end{array}]$$

2c $${P}_{ss}=[\begin{array}{ll}{e}^{i{\beta }_{ss\mathrm{,1}}{L}_{s}} & 0\\ 0 & {e}^{i{\beta }_{ss\mathrm{,2}}{L}_{s}}\end{array}],\,{P}_{cs}=[\begin{array}{ll}{e}^{i{\beta }_{cs\mathrm{,1}}{L}_{c}} & 0\\ 0 & {e}^{i{\beta }_{cs\mathrm{,2}}{L}_{c}}\end{array}]$$

2d $$\langle {\hat{e}}_{i},{\hat{e}}_{j}\rangle ={[Re\{\frac{\int {\vec{E}}_{j}\times {\vec{H}}_{i}^{\ast }\cdot \vec{d}A\int {\vec{E}}_{i}\times {\vec{H}}_{j}^{\ast }\cdot \vec{d}A}{\int {\vec{E}}_{i}\times {\vec{H}}_{i}^{\ast }\cdot \vec{d}A\int {\vec{E}}_{j}\times {\vec{H}}_{j}^{\ast }\cdot \vec{d}A}\}]}^{\mathrm{1/2}}$$

where *C*_*ss,s*_ is the change of basis matrix from isolated waveguide modes *s* to straight section modes *ss* and *C*_*cs,ss*_ is the change of basis matrix from straight section modes to curved section modes *cs*. *θ* and *η* take values of ±1 depending on the relative phases of second mode in the original basis and the new basis: ±1 if they are in phase and −1 if they are out of phase. *P*_*ss*_ and *P*_*cs*_ are the propagation matrices of the straight and curved sections, and contain the phase shifts of the modes as determined by the effective length of the straight and curved sections *L*_*s*_ and *L*_*c*_ and the propagation constants *β*_*x*_. $\langle {\hat{e}}_{i},{\hat{e}}_{j}\rangle$ is the amplitude coupling from mode ${\hat{e}}_{i}$ to ${\hat{e}}_{j}$. In the above equations, we only account for the first two supermodes of the system, which is sufficiently accurate if the individual isolated waveguides are single mode. We also assume no coupling between quasi-TE and quasi-TM modes and negligible back reflection from mode mismatch at the transitions. More sophisticated mode matching methods can account for reflected and radiated modes, but are not necessary for our case^25^.

**Figure 2:**
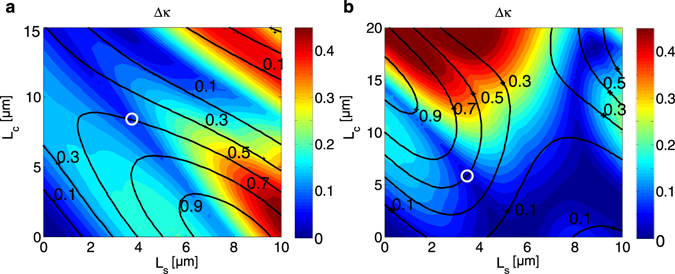
Maximum cross coupled power deviation Δ*κ* versus straight (*L*_*s*_) and curved (*L*_*c*_) lengths of curved directional coupler for (**a**) *R*_*c*_ = 26 *μ*m, *g* = 200 nm, *w*_1_ = *w*_2_ = 400 nm, (**b**) *R*_*c*_ = 13 *μ*m, *g* = 200 nm, *w*_1_ = 380 nm, *w*_2_ = 420 nm. The black contour overlay indicates the cross port power *κ* at 1570 nm. The white circles indicate chosen dimensions for a wavelength insensitive 50/50 coupler.

From Eq. 2(a), the bar port power is *t* = |*A*_1,*out*_|^2^ and cross port power is *κ* = |*A*_2,*out*_|^2^. The wavelength sensitivity of the 50/50 coupler can be quantified using the maximum cross coupled power deviation

3 $$\Delta \kappa ={|\kappa ({\lambda }_{0})-\kappa (\lambda )|}_{max}\approx {|0.5-\kappa (\lambda )|}_{max}$$

over the desired operating wavelength range. *λ*_0_ is the central operating wavelength at which the cross coupled power *κ*(*λ*_0_) is designed to be near 0.5. Figure 2(a) shows the calculated output deviation Δ*κ* for different lengths of *L*_*s*_ and *L*_*c*_ for a curved coupler with radius *R*_1_ = 26.3 *μm*, gap *g* = 200 nm, waveguide widths of *w*_1_ = *w*_2_ = 400 nm and waveguide height *h* = 250 nm, over a wavelength range of 1520 nm to 1620 nm using the TE mode. The black contour overlay indicates the cross coupled power *κ* at 1570 nm. The curved coupler radius is chosen to provide an asymmetry such that maximum cross coupled power for the curved section is close to 0.5 (see the left most section of Fig. 2(a)). From the figure, we can see that a wavelength insensitive 50/50 coupler can be constructed by choosing *L*_*s*_ = 3.9 *μ*m and *L*_*c*_ = 8.1 *μ*m. We have verified the results using 3D finite difference time domain (FDTD) and used these dimensions for our broadband coupler design. Note that the *L*_*s*_ calculated is the *effective* length inclusive of the input/output bends coupling contributions.

Similarly, Fig. 2(b) shows Δ*κ* using the TE mode for a radius *R*_1_ = 13.3*μm* and gap *g* = 200 nm. However, in this case the waveguide widths are chosen to be different, with *w*_1_ = 380 nm and *w*_2_ = 420 nm. Here, the curved coupler radius is chosen such that the maximum cross coupled power for the curved section is close to 1 in order to compensate for the asymmetry in the straight sections. A wavelength insensitive 50/50 coupler can then be constructed by choosing *L*_*s*_ = 3.4 *μ*m and *L*_*c*_ = 6.0 *μ*m. This exercise demonstrates that the TMM design methodology is quite general and can be applied to any cross-section, including operation using quasi-TM modes.

## Results

**Figure 3:**
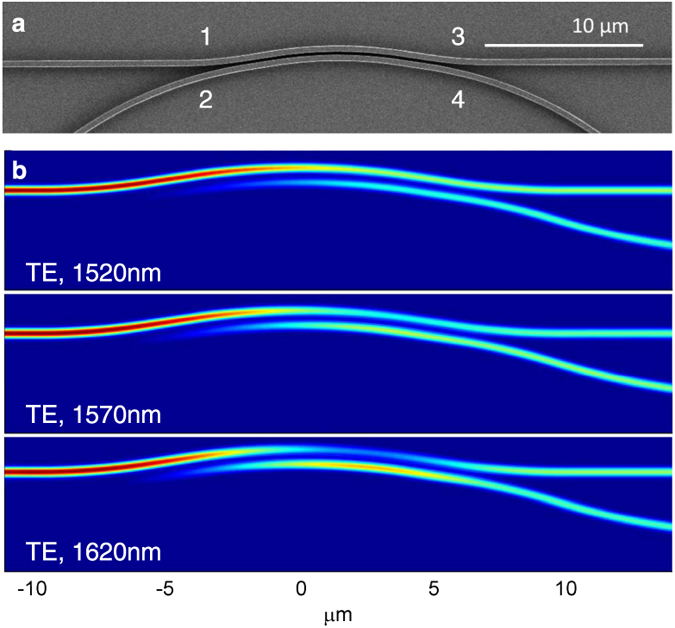
(**a**) SEM image of the fabricated device. (**b**) 3D FDTD simulated power distribution of the designed broadband 50/50 curved coupler for TE mode.

Following Fig. 1, we select *w*_1_ = *w*_2_ = 400 nm, gap *g* = 200 nm, radius *R*_*c*_ = 26 *μ*m. We fabricated the device (see Methods) and the scanning electron microscope (SEM) image is shown in Fig. 3(a). Figure 3(b) shows the calculated device power distribution using 3D FDTD method^24^ for *R*_*c*_ = 26 *μ*m, *L*_*c*_ = 8.1 *μ*m and *L*_*s*_ = 1.8 *μ*m, using the TE mode as input. Here, *L*_*s*_ is the true straight coupler length, as opposed to the *effective* length calculated by the TMM method. For this design layout, we chose *β* = *α* and used a fixed radius for all input/output sections, i.e. all bends have a radius of *R*_2_ except the outer bend in the curved section which is of radius *R*_1_. All the waveguide ends are terminated with inverse tapers. Before characterisation, quasi-TE light was selected with the help of a fibre polariser. An amplified spontaneous emission (ASE) source covering the C- band and L- bands with quasi-TE polarisation was launched into port 1 (as denoted in Fig. 3(a)) of the waveguide using a tapered lensed fibre. The output spectra of the devices at port 3 and port 4 were measured using an optical spectrum analyser. Similarly, port 3 and port 4 were measured with light input from port 2.

**Figure 4:**
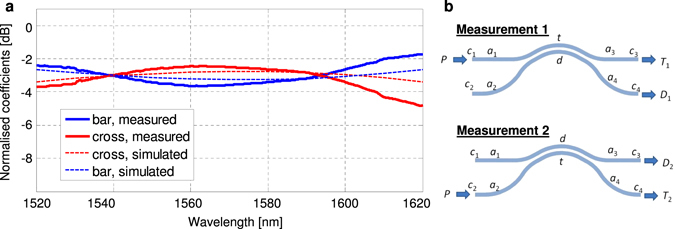
Measured normalised bar and cross coefficients of the fabricated curved directional coupler. The dotted line indicates simulated results as a comparison. (inset) Layout diagram of measurement 1 and measurement 2, indicating different inputs, outputs and sources of loss.

Figure 4 inset shows the schematic of the coupler with the corresponding losses. In measurement 1, we launched the light into port 1 and we measured *T*_1_ and *D*_1_. Similarly, in measurement 2, we launch into port 2 and measured *T*_2_ and *D*_2_. Note that *T* and *D* are interchanged. The four spectra readings obtained consists of other losses such as fibre to waveguide coupling loss and waveguide roughness scattering loss. We want to obtain the normalised bar and cross coupling coefficients. *P* refers to the spectrum of the input ASE source, *c*_*x*_ refers to the fibre to waveguide coupling loss, *a*_*x*_ refers to the waveguide loss, *t* is the bar coefficient, *d* is the cross coefficient of the coupler.

The spectra *T*_1_, *D*_1_, *T*_2_ and *D*_2_ are given by:

4a $${T}_{1}={c}_{1}{a}_{1}t{a}_{3}{c}_{3}P$$

4b $${D}_{1}={c}_{1}{a}_{1}d{a}_{4}{c}_{4}P$$

4c $${T}_{2}={c}_{2}{a}_{2}t{a}_{4}{c}_{4}P$$

4d $${D}_{2}={c}_{2}{a}_{2}d{a}_{3}{c}_{3}P$$

Hence, the normalised bar and cross coefficients are given by:

5a $$\frac{t}{t+d}=\frac{1}{1+\sqrt{\frac{{D}_{1}{D}_{2}}{{T}_{1}{T}_{2}}}}$$

5b $$\frac{d}{t+d}=\frac{1}{1+\sqrt{\frac{{T}_{1}{T}_{2}}{{D}_{1}{D}_{2}}}}$$

The losses such as *c*_*x*_ and *a*_*x*_ will be eliminated. The normalised bar and cross coefficients, *t*/(*t* *+* *d*) and *d*/(*t* + *d*) respectively were calculated and plotted in Fig. 4. The dotted line represents the FDTD simulated spectrum for the bar (blue) and cross (red) port. The bold line represents the experimentally measured data. The device exhibits a wavelength flattened transmission, with a splitting ratio of 3 dB ± 1 dB within a bandwidth of 88 nm. Possible explanations for the difference between simulation and normalised experimental results is the fabrication imperfections and the variance in fibre alignment during the collection of measurement data.

**Figure 5:**
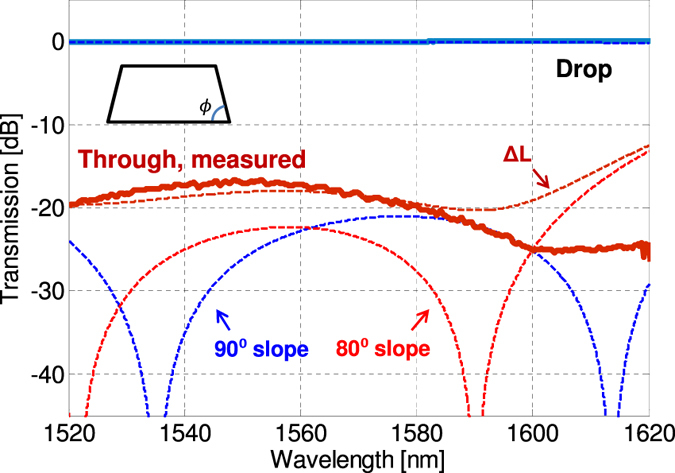
Measured normalised through and drop port powers of the fabricated balanced Mach-Zehnder interferometer. Dashed lines indicate simulations to compare the transmission characteristics of vertical 90° versus non-vertical sidewall slope of 80° and also with a small path length difference of Δ*L*. (inset) Trapezoidal waveguide with sidewall angle of ϕ.

In order to verify that our broadband curved directional coupler design translates well from cleanroom nanofabrication to silicon photonics foundry fabrication, we have fabricated a balanced MZI based on these couplers using A*STAR IME MPW services. As the silicon layer thickness for the MPW is set at 220 nm, the dimensions of the curved directional coupler had to be re-calculated. We chose *R*_*c*_ = 26 *μ*m, gap *g* = 200 nm, waveguide widths of *w*_1_ = *w*_2_ = 420 nm and waveguide height *h* = 220 nm, using the TE mode. The transmission of a balanced MZI is given as

6 $$[\begin{array}{c}T\\ D\end{array}]=M[\begin{array}{ll}{e}^{i\psi } & 0\\ 0 & 1\end{array}]\,M[\begin{array}{c}{A}_{0}\\ 0\end{array}]$$

where *M* is the transfer matrix of the curved coupler as determined by Eq. 2(a). Since for a MZI the transmission is determined by the optical path length difference, there is theoretically no need for the propagation matrix in a balanced MZI. However, due to fabrication imperfections, there will be a small path length difference in the two arms of the MZI which is captured by the phase difference *ψ* = *β*Δ*L*. *T* is the through port amplitude and *D* is the drop port amplitude given a normalised input of *A*_0_. In Fig. 5, we plot the measured normalised transmission of the balanced MZI. The drop port shows high transmission and the through port shows an extinction ratio of >16 dB over 100 nm of bandwidth. It is known that the sidewall slope angle *ϕ* of fabricated waveguides can show a small deviation from vertical. As a comparison, we plot the simulated transmission of such balanced MZI using a sidewall slope angle *ϕ* of 80° and 90°. The effect of non-vertical sidewalls is to shift the centre of the regions of high extinction while still maintaining the wavelength insensitive nature. We note that *ϕ* = 80° is able to match the experimental results slightly better. When a phase of *ψ* = 11° due to path length difference is also included, a reasonable fit is obtained. Active thermo-optic tuning can be used to compensate for the path length difference and further improve the extinction ratio. Other sources of deviation from the simulated transmission characteristics could be the incomplete extinction of TM mode at the input and non-identical couplers at the front and back of the MZI.

Scaled switch fabrics can be constructed by cascading building blocks of 2 × 2 switching elements, each element consisting of a tunable balanced MZI. Using a 2 × 2 switching element with >15 dB extinction over a 75 nm bandwidth, Lee *et al*.^26^ showed scaling to a 4 × 4 switch with crosstalk remaining below −14 dB. However, scaling to 8 × 8 switch caused the crosstalk to grow as large as −5 dB for some switch configurations. Increasing the extinction ratio of the building block 2 × 2 elements is key to enable scaling to larger switch fabrics. Recently, Chen *et al*.^4^ demonstrated a thermo-optic switch with extinction of >20 dB over 140 nm bandwidth that utilises curved directional couplers. However, they used electron-beam lithography and chose a narrow gap of *g* = 110 nm which is difficult to achieve using DUV lithography.

## Discussion

**Figure 6:**
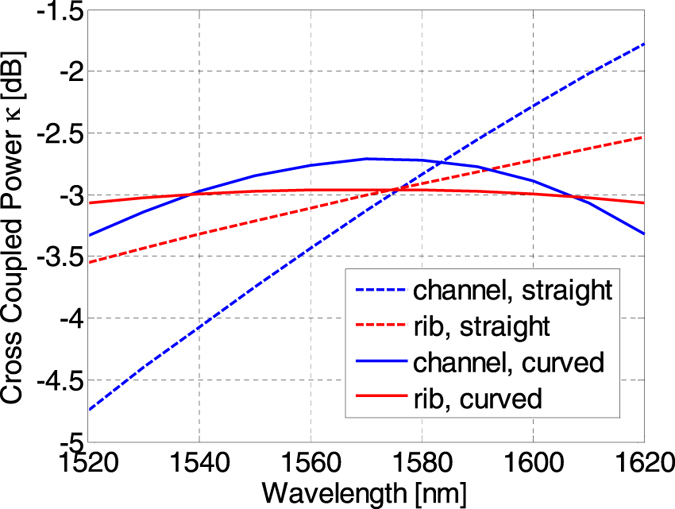
Wavelength dependence of cross coupled power of straight and curved directional couplers using channel and rib waveguide structures.

The TMM method of Eq. 2 and the figure of merit of Eq. 3 provides a methodology to select coupler bending radius *R*_*c*_ and section lengths *L*_*s*_, *L*_*c*_ such as to give a wavelength insensitive splitter. However, there remains several free parameters that have to be chosen prior to the calculation of the various coupler section lengths. These include the waveguide width, height and coupler gap. In general, it will be possible to determine the optimum coupler section lengths according to the figure of merit Eq. 3 for any chosen waveguide cross-section and gap. However, it is pertinent to determine which cross-section would give the better wavelength insensitive response overall. Using coupled-mode theory, it was shown that smaller coupler dispersion can be achieved by having a short coupler (i.e stronger waveguide coupling) and reducing the group index *difference* Δ*n*_*g*_ of the coupler supermodes^27^. For example, we have found that in the SOI platform, rib waveguide couplers have a smaller group index difference compared to channel waveguide couplers. In Fig. 6, we plot the wavelength dependent TE mode cross coupling of conventional straight 50/50 directional couplers and compare them to optimised curved couplers using both channel and rib waveguides. The results were calculated using the TMM method. Although the optimised curved coupler outperforms the corresponding conventional straight coupler, the use of rib waveguides produces a flatter cross coupling overall. For the channel waveguides, we have used *w*_1_ = *w*_2_ = 420 nm, *h* = 220 nm, *R*_*c*_ = 26 *μ*m and *g* = 200 nm. For the rib waveguides, we have used *w*_1_ = *w*_2_ = 400 nm, *h* = 220 nm, *R*_*c*_ = 32*μ*m, *g* = 300 nm and slab height *s* = 120 nm.

In conclusion, we report a general design procedure based on transfer matrix method to construct a broadband 2 × 2 photonic power splitter consisting of a combination of curved and straight coupled waveguide sections. We designed and fabricated such a power splitter with a 50/50 splitting ratio. The device shows a splitting ratio of 3 dB ± 1 dB over a bandwidth of 88 nm centered at 1565 nm, and has a footprint of 20 *μ*m × 3 *μ*m. In addition, we fabricated and measured a balanced MZI consisting of two curved directional couplers using a photonics MPW service showing an extinction ratio of >16 dB over 100 nm of bandwidth. Furthermore, we have investigated the effects of non-vertical sidewalls through simulations and showed that the MZI retains its wavelength insensitive operation. As further exploration, such broadband curved directional couplers may be further generalised to consist of multiple asymmetric sections in the future^28^. We expect such curved directional couplers to be able to find widespread use in multiplexers, splitters, filters and switches in photonic integrated circuits.

## Methods

### Fabrication

Cleanroom nanofabrication techniques are used to fabricate the curved directional coupler device. An SOI wafer (250 nm Silicon on 3000 nm SiO_2_) was patterned using electron-beam lithography, followed by reactive ion etching and plasma enhanced chemical vapor deposition of SiO_2_ as the overcladding. The balanced MZI devices were fabricated using A*STAR IME photonics MPW service.
